# A Case Report of Bilateral Exudative Retinal Detachment in Severe Pre-eclampsia

**DOI:** 10.7759/cureus.56791

**Published:** 2024-03-23

**Authors:** Priya Sivakumaran, Manjiri Khare, Periyasamy Kumar

**Affiliations:** 1 Medicine, Kingston Hospital NHS Foundation Trust, London, GBR; 2 Maternal-Fetal Medicine, University Hospitals of Leicester NHS Trust, Leicester, GBR; 3 Ophthalmology, University Hospitals of Leicester NHS Trust, Leicester, GBR

**Keywords:** acute visual loss, systematic literature review, clinical case report, serous retinal detachment, pre-eclampsia

## Abstract

We report a case of a 31-year-old gravida 2 para 1 female presenting to the optician with a two-week history of blurred vision and persistent headaches at 29 weeks gestation. Visual acuity on presentation was 6/100 in the right eye and 6/24 in the left eye. Fundoscopy of both eyes revealed serous retinal detachment in the absence of background retinal changes. On urgent admission to the maternity assessment unit, blood pressure was 189/126 mmHg and urine dipstick revealed 4+ proteinuria. Due to recurrent poor foetal heart rate variability on cardiotocography monitoring, an emergency caesarean was conducted. Sixteen hours following delivery, visual symptoms had improved, and clinical examination revealed normal blood pressure. An optical coherence tomography scan performed three months later was dry bilaterally with minor retinal pigment epithelium clumping.

Serous retinal detachment involves the separation of the neurosensory retinal layer from the underlying retinal pigment epithelium. It is rare in pre-eclampsia but can be seen in patients with severe disease. The presentation of serous retinal detachment includes acute visual loss, reduced visual acuity, floaters, and flashing lights appearing in the vision. Although alarming on initial presentation, resolution is commonly seen within a couple of days postpartum. The pathogenic mechanism for serous retinal detachment development is widely discussed and thought to include changes to the choroidal circulation. Overall, although often self-resolving, a move to thorough antenatal care and vigilant monitoring in pre-eclamptic women is vital to prevent complications like this from occurring.

## Introduction

Pre-eclampsia is a multisystem hypertensive disorder presenting in women over 20 weeks gestation. It complicates 2-8% of pregnancies, contributing to 15% of pre-term births and between 9% and 26% of maternal deaths globally [1]. Pathophysiology is complex, although endothelial cell dysfunction resulting in poor organ perfusion and uteroplacental hypoperfusion from ineffective spiral artery invasion are discussed in the literature [2,3]. Visual symptoms in pre-eclampsia are described in 25-40% of women and include blurred vision, diplopia, amaurosis fugax, photopsia, scotoma, and homonymous hemianopia [4]. Examples of ophthalmological complications with the disease include hypertensive retinopathy, cortical blindness, retinal vein occlusion, and ischaemic optic neuropathy [5]. Retinal detachment, however, is rare and only affects 0.1-2% of patients with the condition [6]. We describe a case of the development of bilateral serous retinal detachment secondary to severe pre-eclampsia on a background of poor antenatal surveillance secondary to a late booking appointment. This article aims to draw education on the importance of establishing rigid antenatal surveillance, particularly in patients with late booking and complex pregnancy history as well as providing an overview of current advancing evidence in surveillance imaging and how these may be implemented for these high-risk individuals.

## Case presentation

**Figure 1:**
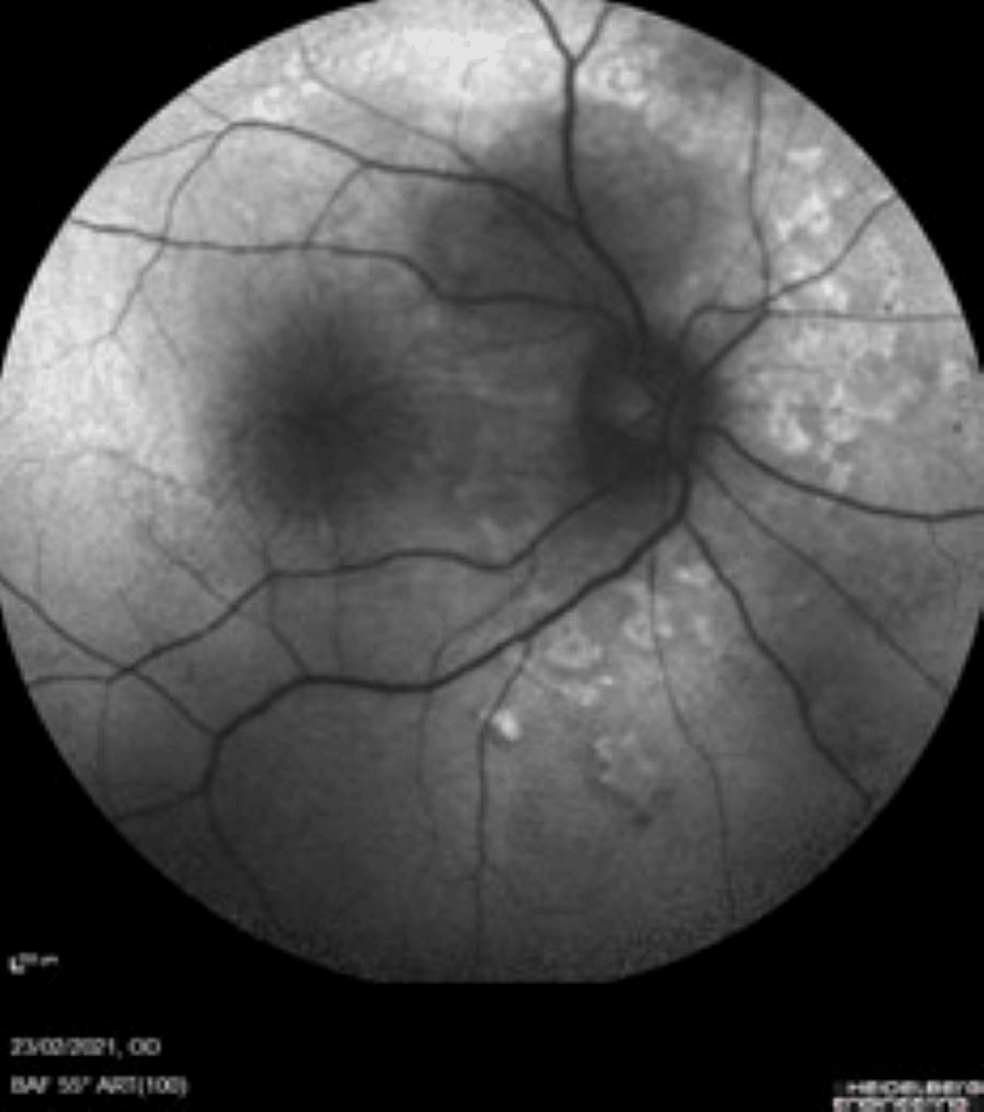
Fundoscopy image highlighting retinal changes in the right eye.

A 31-year-old gravida 2 para 1 female presented to the optician with a two-week history of blurred vision and persistent headaches at 29 weeks gestation in the absence of flashing lights, floaters, and photosensitivity. On initial presentation at eye casualty, an ophthalmological examination revealed a visual acuity of 6/100 in the right eye and 6/24 in the left eye, with no improvement in visual acuity using pinhole bilaterally. Dilated slit lamp examination showed possible cells in the anterior chamber. No relative afferent pupillary defect was present, and intraocular pressure was 16 mmHg and 15 mmHg in the right and left eye, respectively. A dilated fundoscopy of both eyes revealed multi-focal serous retinal detachment in the absence of systemic hypertensive retinal changes. Figure 1 highlights these changes specifically in the right eye.

Her previous pregnancy was a small for gestational-age baby, and she received antenatal care for pre-eclampsia and gestational diabetes throughout. At 36 weeks, she had a vaginal delivery and suffered a fourth-degree tear. Following birth, she continued breastfeeding for two and a half years but was not using contraception. She was unaware she was pregnant again until an ultrasound revealed she was 27 weeks pregnant. Subsequently, a booking appointment was only performed two weeks prior to her presentation, where her blood pressure was 90/60 mmHg and BMI was 26 kg/m2.

An immediate transfer from eye casualty to the maternity assessment unit was performed. Observations on admission showed a blood pressure of 189/126 mmHg, heart rate of 130 bpm, temperature of 37.2°C, respiratory rate of 29/minute, and oxygen saturation of 98% on room air. A urine dipstick test was performed, which showed 4+ proteinuria. Biochemical investigations showed a platelet count of 101 x 10^9/L, albumin of 29 g/L, and a protein creatinine ratio of 2082 mg/g. Neurological examination revealed brisk reflexes.

An urgent infusion of IV magnesium sulphate was commenced, according to the Pritchard regime. A labetalol infusion at 5 mg/hour was also initiated and titrated according to her blood pressure. The maximum dose achieved was 80 mg/hour. Optimisation of preterm delivery included steroids for foetal lung maturity and magnesium sulphate for neuroprotection. Continuous cardiotocography monitoring was commenced and demonstrated poor foetal heart rate variability. Due to recurrent poor foetal heart rate variability on cardiotocography, an emergency caesarean section was performed. A female was delivered at 29 weeks gestation, weighing 1.23 kg. Apgar score was 2 at one minute and 8 at five minutes. Cord blood results highlighted a pH of 7.22, lactate of 3.5, and base excess of -5.

Postpartum, a maintenance dose of IV magnesium sulphate was continued and oral labetalol and nifedipine were started. The placenta was sent for histology and results showed maternal vascular poor perfusion secondary to maternal spiral artery vasculopathy. Sixteen hours postoperatively, her vision improved, and clinical examination revealed normal blood pressure in the absence of proteinuria. An ophthalmological follow-up three months later revealed a quiet anterior segment with a clear lens on eye examination. The posterior segment showed a cup-to-disc ratio of 0.4 in both eyes and an area of retinal pigment epithelial disruption around the macula. An optical coherence tomography scan performed was dry bilaterally with minor retinal pigment epithelium clumping. The patient’s vision had improved significantly, and there was resolution of retinal detachment bilaterally.

## Discussion

Serous retinal detachment is the separation of the neurosensory retina from the underlying retinal pigment epithelium. The pathophysiology of serous retinal detachment development in pre-eclampsia is widely discussed in the literature. Lee et al. suggest endothelial dysfunction and vasospasm induced by severe pre-eclampsia extend more favourably to the choroidal circulation compared to the retinal circulation. This results in occlusion of the choriocapillaris due to fibrinolysis, compromising fluid transport between the retinal pigment epithelium predisposing to fluid accumulation between the retinal layers and subsequent separation of the neurosensory retina from the underlying retinal pigment epithelium [7]. The anatomical course and absent autoregulation of the choroidal circulation are noted by Jayaraj et al. to result in the vulnerability of hypertensive-induced changes in the choroidal capillaries as opposed to the retinal vasculature [8]. Furthermore, fluorescein angiography studies performed by Komoto et al. also support evidence of choroidal circulation involvement through patchy delay in choroidal perfusion within the early phase and fluorescein leakage in the same area in the late phase, with no abnormality seen in retinal perfusion [9]. However, limited studies involving fluorescein angiography in pregnancy are available due to the risks of teratogenicity. Roos et al. also describe intense vasospasm in the choroidal circulation predisposing to retinal detachment. However, they focus on tight junction disruption within the blood-retinal layer, predisposing to increased vascular permeability and subsequent fluid accumulation within the subretinal space [4]. Chen et al. also noted another pathogenic mechanism underlying retinal detachment in pre-eclampsia. They described cytokine release secondary to severe hypertensive disease that mediates an abnormal immunochemical reaction involving calcium ions. Calcium ion involvement in normal regulation and tone of the retinal and choroidal vessels is dysfunctional secondary to this, resulting in subretinal fluid accumulation due to increased vascular permeability [10]. Overall, choroidal circulation dysfunction secondary to intense hypertensive vasospasm, impaired fluid transport across the retinal layers, and subsequent fluid accumulation are significantly reported factors contributing to the pathophysiology of serous retinal detachment in pre-eclampsia [7,4,10].

Two systemic reviews of case reports of retinal detachment in pre-eclampsia by Vigil-De Gracia et al. (2011), who reviewed 28 cases between 1990 and 2010, and Iqra et al. (2022), who extracted data from 17 studies between 2011 and 2021, were reviewed. Out of 28 cases, Vigil-De Gracia et al. found 15 cases were associated with severe pre-eclampsia, 11 with eclampsia or HELLP (haemolysis, elevated liver enzymes, and low platelets) syndrome, and two with eclampsia and HELLP syndrome. Iqra et al. yielded similar figures showing 47% of their cases associated with severe pre-eclampsia and 41% associated with HELLP syndrome. Overall, both reviews noted retinal detachment was more common in nulliparous women and those who delivered via caesarean section with complete resolution in most cases [11,12].

The absence of background hypertensive retinopathy changes in our patient’s fundoscopy is interesting to note given the severity of hypertension on presentation, lack of antenatal monitoring, and control of hypertensive disease throughout her antenatal period. Typically, the effects of systemic hypertension result in changes to both choroidal and retinal vasculature. Choroidal changes include vascular narrowing, vasoconstriction, and extravasation of fluid into the extravascular spaces, and retinal changes include cotton wool spots, intraretinal haemorrhages, and decreased retinal arteriole-to-vein ratio on fundoscopy. The absence of these hypertensive retinopathy changes whilst acknowledging serous retinal detachment as a complication of hypertensive retinopathy and pre-eclampsia is also noted in several case reports [1,9,13]. A study performed by Reddy et al. involving 78 patients with pregnancy-induced hypertension showed many of their patients had grade 1 or grade 2 retinal changes according to the Keith-Wagener hypertensive retinopathy classification system, and no patients had evidence of hard exudates, cotton wool spots, haemorrhages, and optic disc swelling. They suggested these results may be secondary to the early detection of hypertension in their antenatal clinics with early preventative management established at the time of their study. They found statistically significant positive changes in retinal variations and severity of pregnancy-induced hypertension, indicating many of their patients had well-controlled disease secondary to vigilant antenatal monitoring, screening, and preventative measures, resulting in mild hypertensive retinopathy changes [14]. It is difficult, to accept similar implications for the absence of background hypertensive retinopathy changes in our patient due to the lack of antenatal monitoring and preventative measures established. However, there is a possibility that she may have maintained sufficient blood pressure control before her acute admission, meaning only a short history of severe hypertension may have resulted in initial choroidal circulation dysfunction due to it being more susceptible to hypertensive changes compared to the retina.

Difficulty in attaining appropriate monitoring due to late booking as well as a previous complicated pregnancy should have meant a high suspicion of complication development be anticipated in our patient. Hence, extensive assessment equivalent to that in the first trimester should have been implemented and is necessary for women booking further advanced within pregnancy to establish early possible maternal or foetal complications. There is advancing evidence surrounding the use of orbital and Doppler ultrasound in pre-eclampsia diagnosis, surveillance, and identification of complications, which may provide supplementation to current antenatal monitoring in these high-risk individuals. Ferhi et al. outline the diagnostic importance of ultrasound assessment in visual loss with pre-eclampsia using a 7.5 MHz linear probe placed gently on a closed eyelid. Through the positioning of the ultrasound probe and globe of the patient, evaluation of the optic nerve sheath diameter for assessment of intracranial pressure as well as retinal detachment can take place. Emphasis is seen in how most emergency physicians and anaesthesiologists are more comfortable using ultrasound as opposed to performing an ophthalmoscope and fundoscopy assessment [15]. The increasing use of ultrasound imaging in pre-eclampsia assessment is beneficial due to its accuracy, flexibility, economical, and non-invasive nature when compared to additional imaging that may also be used for diagnostic purposes such as CT/MRI brain imaging [15].

The movement towards specific Doppler ultrasound assessment of the maternal ophthalmic artery has also been described by Kane et al. in the effective assessment of complication development in pre-eclampsia. Comparison of maternal ophthalmic artery Doppler assessment to traditional transcranial Doppler deemed it superior for accurate evaluation due to its ability to assess smaller calibre intracranial vessels more vulnerable to cerebrovascular changes that occur in pre-eclampsia, compared to larger calibre vessels that traditional transcranial Doppler was limited to [16]. Onwudiegwu et al. performed a case-control study using 71 pre-eclampsia women and 72 normotensive women, assessing visual acuity, fundoscopy changes, intraocular pressure, and assessment of maternal ophthalmic artery Doppler ultrasound. Results showed that three patients with pre-eclampsia had abnormal fundoscopy changes compared with the control, but all patients had significant differences in end-diastolic velocity and intraocular pressure. The study included a healthy, asymptomatic woman with controlled blood pressure. Evaluation of these results shows maternal ophthalmic artery Doppler assessment offers increased sensitivity in establishing pre-eclamptic complications early when compared to fundoscopy. The conclusion of the study recommended a robust surveillance protocol involving the use of antenatal maternal ophthalmic artery Doppler ultrasound assessment and regular fundoscopy in women diagnosed with pre-eclampsia to help mitigate the associated complications [17]. Conduction of further studies using ultrasound assessment in pre-eclampsia is essential to draw definitive conclusions on effectiveness and influence on maternal and foetal outcomes.

Serous retinal detachment typically self-resolves within a couple of days to weeks in patients with pre-eclampsia. A poor prognostic outcome is noted in patients with macula involvement, extensive retinal pigment epithelial necrosis, and the presence of another retinopathy [8].

## Conclusions

To conclude, the development of serous retinal detachment in pre-eclampsia is rare, but often self-resolving if detected early. Strict antenatal management with surveillance tools, including the advancement of Doppler ultrasound assessment of the maternal ophthalmic artery, is vital in pre-eclampsia complication screening and prevention. Although an obstetric condition, pre-eclampsia can present with multisystem complications, therefore vigilant detection and prompt treatment are the responsibility of all specialities and multidisciplinary members. It should not be seen as a segmental duty for obstetrics alone.
